# Renal Torpidity

**Published:** 1891-05-16

**Authors:** 


### RENAL TORPIDITY.

"Torpidity of the liver" has long been associated in the minds of the public and of the profession with a number of more or less definite symptoms, but renal torpidity is a new term employed by Dr. H. A. Hare for a condition that he regards as arising from a corresponding functional inactivity of the kidneys. This condition of renal atrophy has proved to be the cause of many cases of much distress and minor ill-health. Dr. Hare, writing in the *University Med. Magazine*, affirms that if we admit that the kidneys do temporarily become inactive at any time, then their importance as excretory organs at once shows that such a condition must be followed by the retention of effete materials and the development of symptoms of a more or less active character. We are all on the *qui vive* for cardiac disorders from indigestion, or for headache and languor due to hepatic troubles, but if none of these are found to exist we are often at a loss to determine the cause of the ailment. For a number of months the author has been interested in the study of several cases in which the kidneys seemed to be rendered inactive or deranged by disorders of the digestive functions, or in which the digestive functions seemed impaired by primary renal torpidity. In some cases this torpidity consisted not in an inability on the part of the kidneys to carry on ordinary effort, but in a slowness of action when calls beyond their regular duties were laid upon them. The following instance illustrates those cases in which the digestive disorder seemed to cause not only inactivity of secretion, but, in addition, albuminuria: "C. H., female, aged three, appears to be perfectly healthy and well nourished. Is not fretful in the day, nor restless at night; appetite good. Six months ago, while ailing slightly, the parents noticed that the urine was very concentrated and of a very dark colour. When examined by a physician it was found to be heavily loaded with albumen. Subsequently, after treatment, the albuminuria disappeared and did not reappear till an attack of gastro-intestinal catarrh of a mild type came on, preceded by a few days of constipation, which soon merged into a mucous diarrhœa, the passages being very fœtid, and containing undigested food. All attempts to check the diarrhœa failed until by the use of alkaline diuretics the urine became clear and almost free from albumen, when the diarrhœa also ceased spontaneously." This case is cited by the author as indicating that the kidneys may be, as already indicated, capable of doing ordinary work, but fail if by disturbances in the system they are required to eliminate any irritating products formed by decomposing food in the intestine. The next case typifies another form in which the renal torpidity causes a series of unpleasant symptoms, followed by marked disturbances of digestion. "P.H., aged 29, a male, apparently in perfect health, but much confined to his desk, suffers from frequent attacks of headache and indigestion. The headache follows a feeling of languor and irritability, which sometimes lasts several days. On the day on which the attack culminates he notices about ten o'clock in the morning a feeling of great sleepiness, while slowness of thought becomes so marked as to render conversation difficult; sometimes amnesic aphasia is present to a very slight extent. These symptoms are rapidly followed by a very severe headache. Cold feet, more or less nausea, but no vomiting unless this is provoked by tickling the fauces. If vomiting is produced, however, butyric acid is sometimes present in large amounts. During this time there is apparently no secretion of urine, but after three or four hours a sensation of fullness about the neck of the bladder comes on, which is partly relieved by the passage of a few drops of concentrated urine, and soon after this urinary secretion becomes profuse, the headache ceases, and the urine when passed is limpid and of a very low specific gravity, but scalds the urethra very severely. These attacks come on irrespective of diet or manner of living, and are always preceded for several days by the secretion of a concentrated urine of decided odour. Further, they can always be prevented if the patient drinks large amounts of Vichy or other diuretic waters as soon as he notices an increased specific gravity in his urine." This case illustrates a renal torpidity overcome by renal stimulants.

